# Implementing a guideline for the treatment of type 2 diabetics: results of a Cluster- Randomized Controlled Trial (C-RCT)

**DOI:** 10.1186/1472-6963-7-79

**Published:** 2007-06-04

**Authors:** Carla Perria, Donatella Mandolini, Carmelina Guerrera, Tom Jefferson, Paolo Billi, Virgilio Calzini, Alfonso Fiorillo, Giuseppe Grasso, Sergio Leotta, Walter Marrocco, Concetta Suraci, Amina Pasquarella

**Affiliations:** 1 Community Health Unit, Lazio Region Public Health Agency, Rome, Italy; 2 Cochrane Vaccine Field, Cochrane Collaboration, Rome, Italy; 3 Lazio Region Family Practice Training School, Rome, Italy; 4 Diabetes Centre, Sandro Pertini Hospital, Rome, Italy

## Abstract

**Background:**

In Italy many diabetics still lack adequate care in general practice. We assessed the effectiveness of different strategies for the implementation of an evidence-based guideline for the management of non-complicated type 2 diabetes among General Practitioners (GPs) of Lazio region.

**Methods:**

Three-arm cluster-randomised controlled trial with GPs as units of randomisation (clusters). 252 GPs were randomised either to an active strategy (training module with administration of the guideline), or to a passive dissemination (administration of the guideline only), or to usual care (control). Data on prescriptions of tests and drugs were collected by existing information systems, whereas patients' data came from GPs' databases. Process outcomes were measured at the cluster level one year after the intervention. Primary outcomes concerned the measurement of glycosilated haemoglobin and the commissioning of micro- and macrovascular complications assessment tests. In order to assess the physicians' drug prescribing behaviour secondary outcomes were also calculated.

**Results:**

GPs identified 6395 uncomplicated type 2 patients with a high prevalence of cardiovascular risk factors. Data on GPs baseline performance show low proportions of glycosilated haemoglobin assessments. Results of the C-RCT analysis indicate that the active implementation strategy was ineffective relating to all primary outcomes (respectively, OR 1.06 [95% IC: 0.76–1.46]; OR 1.07 [95% IC: 0.80–1.43]; OR 1.4 [95% IC:0.91–2.16]. Similarly, passive dissemination of the guideline showed no effect.

**Conclusion:**

In our region compliance of GPs with guidelines was not enhanced by a structured learning programme. Implementation through organizational measures appears to be essential to induce behavioural changes.

**Trial registration:**

ISRCTN80116232

## Background

In Italy diabetes mellitus (DM) is a major health problem with a prevalence of 3–4% [1-3] and considerable resources are committed to addressing treatment of micro- and macrovascular complications [4]. In Italy, patients with type 2 non-complicated DM are mainly treated in general practice, whereas patients with type 1 DM and complicated patients usually receive specialist care. Data from an unpublished survey, conducted in 2003 in a local health district of Lazio, showed that the overall quality of DM care often fails to achieve established standards, thus needing urgent intervention to raise standards and protect those most vulnerable from the consequences of DM. Strategies for changing general practitioner's (GPs) behaviour by dissemination and implementation of guidelines and translating evidence into practice are notoriously difficult areas [5-7]. A large systematic review by Grimshaw et al concluded that there is an imperfect evidence base to support decisions on which strategy is likely to be effective [8]. Further uncertainties concern knowledge on service organisation and delivery of diabetic care and the lack of comparative studies on the effects of guideline implementation strategies in Italy.

We report on a cluster-randomised trial (C-RCT) to assess the effects of two different strategies of introducing an evidence-based guideline for the treatment of type 2 DM in primary care. The C-RCT design (randomisation at the level of professional practice or health care organization) represents the optimal design when evaluating dissemination and implementation strategies [9]. As systematic reviews can inform and contribute to the correct design of randomised controlled trials, our protocol was based on three Cochrane and one UK Health Technology Assessment Programme systematic reviews of guideline dissemination and implementation strategies [10-12].

## Methods

### Participants

Eligible study participants were GPs taking part in an electronically-linked disease surveillance network and receiving a specific fee for computer-made prescriptions; they were about 50% of the whole population of Lazio GPs, and on average younger than the non-using informative instruments in their clinical practice. In order to minimize the risk of contamination due to GPs working in associated forms, we used a software which assigned a zero probability to be selected to members of the same practice, once one of them has already been extracted. Participants were recruited during the month of December 2003 and follow-up took place from January 2004 to December 2004.

Recruitment was performed by an invitation letter written in a standardized format, giving information about the study project. Every GP represented a cluster. The study was carried out in the primary care setting of Italian National Health Service in the Lazio region of Central Italy, and was focused on the management of patients with non-complicated type-2 DM, defined as diabetes disease in absence of even only one of the following conditions: ocular complications (retinopathy, cataract, or glaucoma); renal complications (nephropathy, chronic renal failure); neurological complications (autonomic neuropathy, peripheral neuropathy); diabetic foot (deformity, Charcot foot, infection, ulcer, gangrene, amputation); TIA/ictus; obstructive disease of lower limbs.

In our design, GPs were "units of measure" (clusters) of the process of diabetic care delivery, as they managed laboratory and preventive care for most patients with type 2 DM. They were asked to identify their patients accordingly to the above case definition and in observance with the following exclusion criteria: age < 40 years; gestational diabetes; diabetes in pregnancy.

Two different data sources were used. Patient descriptive data (baseline information) were transmitted on-line by GP participants who were given a login and a password to access personal web pages. Data on prescriptions of drugs, tests and outpatient appointment visits were extracted by current information systems, which routinely assemble data for reimbursement purposes, in particular the Regional Drug Information System, which collects all prescriptions of drugs reimbursed by the Italian NHS, and the Ambulatory Care Information System, which gathers all prescriptions of diagnostic tests and outpatient visits.

Data on the process of care for DM were collected during the 12 months of follow-up after the intervention (year 2004) and main outcomes were constructed and assessed from GPs' prescribing and commissioning behaviour.

### Interventions

The intervention was structured learning and distribution of the guideline to participant GPs (clusters).

#### Guideline choice

In order to choose an appropriate guideline we examined the methodological issues on guideline evaluation and implementation strategies and we conducted a systematic search and evaluation of existing guidelines on diabetes care [13-15]. We used the quality assessment criteria and the quality assurance scores of the National Guideline Program of the Italian Institute of Health [16] and we performed a qualitative assessment of guidelines applicability to our regional context. We chose a French guideline [17], which we translated, updated and adapted for Italian GPs.

#### The intervention program

We structured the intervention program through a process of identification of barriers to implementation of recommendations and factors that may facilitate changing professional behaviour, together with an estimation of the resources available within the governance budget. We developed the intervention to be preferably a single and not a multi-faceted intervention, to be easy to carry out and to be reproducible in the implementation of other guidelines. The intervention program included either a two-day training course with CME credits and dissemination of the guideline (arm 1, active implementation) or dissemination of the guideline without the training course but with a written request to implement the guideline (arm 2, passive dissemination). The control group represented the usual care. The training course was organized as parallel sessions of teaching modules together with interactive and group work sessions with discussion of the content of the guideline. An entry questionnaire was given to participants to obtain a picture of current care for diabetic patients.

### Objectives

The primary objective of the study was to assess the effectiveness of different strategies for the implementation of an evidence-based guideline for the management of non-complicated type 2 DM among GPs of the Lazio region. Our null hypothesis was that a structured intervention would be no more effective than a passive dissemination of the guideline or of a no-intervention strategy. The secondary objective was to estimate the efficiency of resource use associated with the intervention through a cost-effectiveness analysis. Both the objectives were to be assessed at cluster level.

### Outcomes

Process of care variables were considered study outcomes, all aimed at assessing physician-changing behaviour for the 12 months following the intervention, by using data from existing information systems. As Lazio GPs are not trained to register clinical data for epidemiological purposes, patient's health outcomes were not collected. Outcomes were expressed as proportions of patients who were prescribed requests for tests, for outpatient appointment visits and drugs in the follow-up year. All outcomes were evaluated at the patient level and aggregated to represent clusters.

#### Primary outcomes

We evaluated GPs' adherence to guideline recommendations for diabetes management by the following assessments: assessment of glycaemic control: we chose as primary outcome the measurement of glycosilated haemoglobin, as the proportion of patients who were prescribed 3 measurements of glycosilated haemoglobin with at least two months' interval per year; assessment of macrovascular complications aimed at early detection of the risk of macrovascular complications of DM: we chose as primary outcome the proportion of patients who were prescribed all macrovascular complications assessment tests (ECG and complete lipid profile, i.e. total cholesterol, HDL cholesterol and tryglicerides simultaneously) per year; assessment of microvascular complications aimed at early detection of the risk of microvascular complications of DM: we chose as primary outcome the proportion of patients who were prescribed all microvascular complications assessment tests (eye examination or fundus and blood creatinine or creatinine clearance and microalbuminuria) per year.

#### Secondary outcomes

To explore participant GPs' drug prescribing behaviour we also calculated secondary outcomes, as indicators on drugs prescribed for the management of the disease and of correlated cardiovascular risk factors: pharmacological management of DM, assessed by the proportion of overweight/obese diabetics who were prescribed methformin in monotherapy as first-choice therapy and by the proportion of elderly diabetics (>70 years) who were prescribed long-life sulphonylureas (contraindicated in old age for their long biological action which increases the risk of hypoglycaemia); pharmacological management of cardiovascular risk factors, assessed by the proportion of diabetics with at least 1 cardiovascular risk factor who were prescribed anti-platelet drugs and by the proportion of hypertensive diabetics who were prescribed first-choice antihypertensive drugs in monotherapy; the proportion of patients with dyslipidemia receiving lipid-lowering-drugs.

For pharmacological indicators, patients were considered as previously untreated if they had no medication prescriptions recorded in the 12 months prior to the beginning of the trial.

### Sample size

The sample size calculation [18] took into account the intra-cluster correlation coefficient, the number of events, the expected effect, and the power of the study. On the basis of the unpublished survey we assumed an intracluster correlation of ρ = 0.1 [19], an average of 15 diabetic patients for each GP, and a worst-case control rate of 50%. Under these assumptions we anticipated a power of 90% to detect a difference of 10% in rates between one arm and the other with α = 0.05 with 84 GP for each group. The total sample size was 252 GPs.

### Randomisation

#### Sequence generation

Our randomisation sequences was computer-generated. GPs who accepted to take part in the study, were assigned by simple random allocation by the REXSCO [21] software, which assigns to same-practice partners a nil probability of being randomised, thus minimising the chances of participant contamination. Randomisation was performed by a researcher not involved in the study and who was blind to the identity of the practices.

#### Allocation concealment

Given the cluster design participant concealment was not possible but data extraction and analysis was carried out in blind fashion by one of us (DM).

### Statistical methods

We carried out comparisons of primary outcomes between the intervention and control arms at cluster (GP) level as this was the unit of randomisation [22] and data were analysed using a cluster specific method, Generalised Estimating Equation (GEE) model [23,24].

## Results

### Participant flow

**Figure 1:**
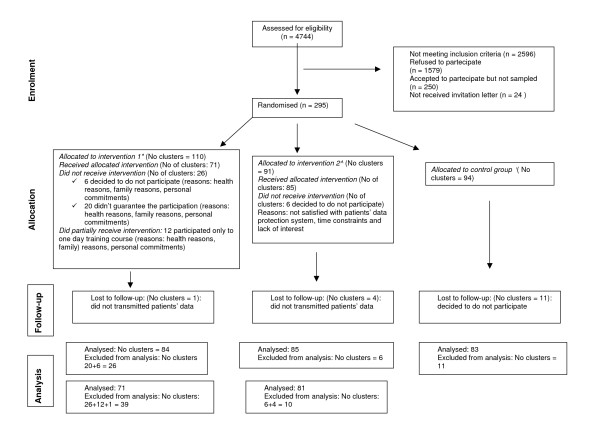
Flow diagram of the progress of clusters. Legend: *: Two-day training course + CME credits. ^ : Passive dissemination of the guideline. °: No intervention group, continuing current practice.

A flow-chart of the progress of clusters through the phases of the trial is at Figure 1. We wrote to 2148 of the 4744 Lazio GPs who were known to be computer-literate because they were in receipt of a computer allowance and were users of regional surveillance software. Twenty five percent (545) of eligible GPs agreed to take part (potential participants). Of these we randomly assigned 84 "first choices" and 36 "stand-ins" for each arm. As 43 GPs declined to take further part after they had been notified of the outcome of randomisation, we contacted a total of 295 potential participants (110 in arm 1, 91 in arm 2 and 94 in arm 3). Post-randomisation attrition was highest in arm 1: 26 (60%) vs 6 (14%) vs 11 (26%) probably because of the requirement to attend our course.

### Baseline data

Tables 1 and 2 show the distribution of characteristics of (respectively) participant GPs (clusters) and diabetic patients cared by GPs (individuals). Baseline information was available for 6290/6395 patients.

**Table 1:** Baseline information on GP participants (clusters)

**Item**	**Active implementation**	**Passive dissemination**	**Usual care**	**Total**
No of participant GPs	84	85	83	252
Mean age (SD) (in years)	48.7* (4.3)	50.6 (4.9)	50.7 (4.8)	50.0 (4.7)
No (%) by gender M	69 (82)	72 (85)	72 (87)	213 (85)
F	15 (18)	13 (15)	11 (13)	39 (15)
Mean (SD) seniority (in years)	14.6 (6.3)	16.3 (7.0)	16.8 (6.8)	15.9 (6.8)
No (%) of GPs with <500 patients	9 (11)	4 (5)	9 (11)	22 (9)
No (%) of GPs with 500–1000 patients	16 (19)	18 (21)	11 (13)	45 (18)
No (%) of GPs with >1000 patients	59 (70)	63 (74)	63 (76)	185 (73)
No of diabetic patients	1973	2190	2232	6395
Mean (SD) diabetic patients per GP	23.5 (13.0)	25.8 (14.7)	26.9 (17.5)	25.4 (15.1)
No (%) of singleton GPs	21 (25)	18 (21)	13 (16)	52 (21)
No (%) in group practices	63 (75)	67 (79)	70 (84)	200 (79)
No (%) of GPs practicing in Rome	47 (56)	49 (58)	41 (49)	137 (54)
No (%) of GPs practicing in suburbs of Rome	14 (17)	13 (15)	15 (18)	42 (17)
No (%) of GPs practicing elsewhere in Lazio	23 (27)	23 (27)	27 (33)	73 (29)

*p < 0.017 = p_Bonferroni_

**Table 2:** Baseline information on patients for whom information was available (6290/6395) cared by GP participants

**Item**		**Active implementation No (%)**	**Passive dissemination No (%)**	**Usual care No (%)**
Gender	F	971(50)	991(47)	1060(47)
	M	981(50)	1115(53)	1172(53)
	total	1952	2106	2232
Age (years)	<50	139 (7)	119 (6)	128 (6)
	50–70	1089 (56)	1155 (55)	1279 (57)
	>70	724 (37)	832 (39)	825 (37)
Patient managed by	GP only	801 (41)	936 (44)	1056 (47)
	GP with specialist	1130 (58)	1133 (54)	1115 (50)
	Integrated care	21 (1)	37 (2)	61 (3)
Diabetes duration (years)	<5	691 (35)	673 (32)	781 (35)
	5–10	718 (37)	778 (37)	803 (36)
	11–20	364 (19)	479 (23)	456 (20)
	>20	80 (4)	126 (6)	157 (7)
	Not known	99 (5)	50 (2)	35 (2)
Glycaemic control	Optimal	457 (23)	571 (27)	495 (22)
	Acceptable	1005 (52)	1111 (53)	1140 (51)
	Inadequate	331 (17)	362 (17)	374 (17)
	Not known	159 (8)	62 (3)	223 (10)
Therapy	Diet only	319 (16)	254 (12)	358 (16)
	Pharmacological	1633 (84)	1852(88)	1874(84)
Hypertension	Yes	1183 (61)	1334 (63)	1334 (60)
	No	748 (38)	741 (35)	864 (39)
	Not known	21 (1)	31 (2)	34 (1)
Hypercolesterolemia	Yes	802 (41)	952 (45)	1037 (47)
	No	1066 (55)	1085 (52)	1099 (49)
	Not known	84 (4)	69 (3)	96 (4)
Smoking	Yes	336 (17)	416 (20)	400 (18)
	No	1494 (77)	1597 (76)	1672 (75)
	Not known	122 (6)	93 (4)	160 (7)
BMI	Normal (<25)	351 (18)	374 (18)	365 (16)
	Overweight (25–29)	628 (32)	726 (35)	771 (35)
	Obese (>29)	307 (16)	366 (17)	370 (17)
	Not known	666(34)	640 (30)	726 (33)

### Numbers analysed

We carried out two analyses, one by including all 252 participant GPs (84 in arm 1, 85 in arm 2 and 83 in arm 3) who had identified their DM type 2 patients and the second, per protocol analysis (PPA), on the 235 participants who had identified their patients, provided their baselines characteristics and taken part in the full course (arm 1 only) (71 in arm 1, 81 in arm 2 and 83 in arm 3).

### Outcomes and estimation

Table 3 shows pre-intervention (year 2003) and post-intervention (year 2004) values of primary outcomes together with their individual components and change over time for each of the 3 arms.

**Table 3:** Pre-intervention (year 2003) and post-intervention (year 2004) values of primary outcomes, their individual components (*in brackets*) and change over time for each of the 3 arms

**Primary outcomes **(Individual components)	**Arm**	**Pre-intervention value %**	**Post-intervention value %**	**Δ%**
**Metabolic control**	Active implementation	11,0 (218/1973)	11,9 (235/1973)	7,8
	Passive dissemination	7,7 (169/2190)	10,1 (222/2190)	31,4
	Usual care	8,8 (196/2232)	10,3 (230/2232)	17,3

**Macrovascular complications**	Active implementation	12,7 (250/1973)	14,0 (277/1973)	10,8
	Passive dissemination	10,7 (235/2190)	11,7 (257/2190)	9,4
	Usual care	10,9 (244/2232)	12,4 (277/2232)	13,5
(ECG)	Active implementation	26,1 (515/1973)	26,0 (513/1973)	-0,4
	Passive dissemination	25,9 (568/2190)	25,0 (547/2190)	-3,7
	Usual care	24,9 (555/2232)	24,2 (541/2232)	-2,5
(Lipid profile)	Active implementation	30,8 (608/1973)	35,7 (705/1973)	16,0
	Passive dissemination	25,7 (563/2190)	28,5 (624/2190)	10,8
	Usual care	25,0 (559/2232)	30,3 (677/2232)	21,1

**Microvascular complications**	Active implementation	5,3 (104/1973)	6,9 (136/1973)	30,8
	Passive dissemination	4,5 (98/2190)	4,9 (108/2190)	10,2
	Usual care	5,0 (112/2232)	4,7 (105/2232)	-6,3
(Retinal screening)	Active implementation	25,0 (494/1973)	26,6 (525/1973)	6,3
	Passive dissemination	24,2 (530/2190)	23,9 (523/2190)	-1,3
	Usual care	22,9 (512/2232)	22,7 (507/2232)	-1,0
(Microalbuminuria)	Active implementation	10,5 (208/1973)	13,7 (271/1973)	30,3
	Passive dissemination	8,7 (191/2190)	10,5 (229/2190)	19,9
	Usual care	9,8 (219/2232)	11,9 (265/2232)	21,0
(Creatinine serum level)	Active implementation	54,6 (1078/1973)	54,2 (1070/1973)	-0,7
	Passive dissemination	48,7 (1067/2190)	51,6 (1129/2190)	5,8
	Usual care	47,1 (1052/2232)	50,4 (1124/2232)	6,8

C-RCT results obtained processing data from all 252 clusters are shown in Table 4.

**Table 4:** Results of C-RCT analysis (year 2004) performed on all 252 GPs recruited

**Principal indicator**	**Arm**	**Value**	**ICC**	**OR**	**95% CI**
Metabolic control	Active implementation	11,9 %	0,069	1,06	(0,76 – 1,46)
	Passive dissemination	10,1 %		0,93	(0,67 – 1,30)
	Usual care	10,3 %		1	
Macrovascular complications	Active implementation	14,0 %	0,054	1,07	(0,80 – 1,43)
	Passive dissemination	11,7 %		0,93	(0,70 – 1,24)
	Usual care	12,4 %		1	
Microvascular complications	Active implementation	6,9 %	0,046	1,4	(0,91 – 2,16)
	Passive dissemination	4,9 %		1,11	(0,73 – 1,69)
	Usual care	4,7 %		1	

ICC = intracluster correlation coefficient; OR = odds ratio; 95% CI = confidence interval

#### Active implementation *vs *usual care

Analyses of data measuring the impact of the active strategy shows that there was no effect either on the metabolic control indicator (OR 1,06 [0,76–1,46]), or on macrovascular complications assessment tests (OR 1,07 [0,80–1,43]) or on microvascular complications assessment tests (OR 1,4 [0,91–2,16]).

#### Passive dissemination *vs *usual care

Analyses of data measuring the effect of passive dissemination of the guideline shows similar results for all the three primary outcomes, respectively OR 0,93 [0,67–1,30] for the metabolic assessment, OR 0,93 [0,70–1,24] for the macrovascular complications assessment test and OR 1,11 [0,73–1,69] for the microvascular complications assessment test.

Per protocol analyses gives similar results (*data not shown*). On the basis of analyses of per protocol data and of data relating to all 252 clusters we cannot reject our null hypothesis, as our findings show an absence of correlation between active or passive educational training of GPs and physicians attitude at following the indications of the guideline. As results showed the non-effectiveness of the intervention strategy, we did not perform any economic evaluation or carry out analysis on participant sub-clusters.

## Discussion and Conclusion

Our trial did not show significative differences between arms in the effectiveness of two different strategies to implement a good quality guideline for caring for people with type 2 uncomplicated diabetes. The dissemination of our guideline and a specifically targeted educational program seemed to unable to persuade GPs to change their current clinical practice. Although disappointing to us, these findings represent the first experimental evidence of the problems of putting evidence into practice available in Italy. They suggest that at both regional and national level there is an urgent requirement to attempt complex and articulated paths in an effort to increase quality of care. There is a growing body of evidence about the importance of an existing definite organizational framework as the basis for any changing behaviour by health care providers [25,26] and a recent systematic review highlights a substantial improvement in patient glycaemic control using case management [27]. Disease management models have been recently introduced in some regions, but although some pilot studies have shown promise [28], substantial differences between regional contexts may make these results not generally applicable. In Lazio many community services are performed by hospital outpatient departments with scarce integration between hospital and community and between primary and secondary care. This lack of integration is reflected in patchy information exchange between operators. An important issue is the generalisability (or external validity) of the trial's findings at the cluster (GP) level. Available systematic reviews [7-9] reported possible non-specific effects on the control arms of C-RCTs which could hinder generalisability of results. In Lazio there are very scarce data on knowledge and perception of guidelines and commonly GPs have difficulty in being involved in institutional programs for improving the quality of care. For a number of reasons [29,30]. Internal barriers include time constraints, possibly inadequate reimbursement and disagreement with innovative programs. External barriers include individual patient needs, limited systems to support chronic disease management and poor patient adherence to treatment. In addition a large part of GPs may not be accustomed to web-based data loading and may not be accustomed to taking part in trials. It is possible that the performance of the control group (current practice arm) could have been different from other current practice with unconscious improvement from the beginning of the trial when control participants were asked to define and transmit a set of data relating to their DM patients. This may have had an impact on the applicability of our results. Conversely some contamination with participants belonging to different arms cannot be discounted, although we have no proof of its occurrence. The conclusions of our study are not very different from those reported by a survey conducted in 2004 by the Italian Institute of Health on a population of diabetics sampled in all Italian regions, which reported scarce adherence to clinical guidelines either by GPs or by specialists and large opportunities for improvement of care [31]. Evidence from systematic reviews is at present unable to point to the most effective way of changing doctors' behaviour because of poor methodological quality of the majority of the included studies (major recurring problems were unit of analysis errors, baseline imbalance and within-group rather than between groups comparisons). In addition most studies used process measures for their primary end-point (like we did) and real improvements in health remained an issue of debate. In conclusion the effectiveness of implementation strategies is strongly correlated to the local context and could largely vary under different circumstances. Efforts should be directed to increase the body of evidence with more and better quality comparative studies. At the same time, policy makers have the responsibility to administrate the limited resources for clinical governance on the basis of a thorough knowledge of settings and problems.

The following are apparent limits of trial: uncertain representativeness of enrolled GPs compared to the remaining GPs which could affect external validity of our results; better performance of enrolled GPs; non-attendance for teaching sessions rate (13 out of 84, 15%).

## Competing interests

The author(s) declare that they have no competing interests.

## Authors' contributions

AP, CP, PB, CG, TJ, SL, CS, AF WM and VC wrote the protocol, chose and adapted the guideline. DM wrote the statistical methods and carried out data analysis. All authors contributed to data interpretation and inputted to the final version of the manuscript which was written by CP, DM and TJ.

## Pre-publication history

The pre-publication history for this paper can be accessed here:


